# Detection and tracking of overlapping cell nuclei for large scale mitosis analyses

**DOI:** 10.1186/s12859-016-1030-9

**Published:** 2016-04-26

**Authors:** Yingbo Li, France Rose, Florencia di Pietro, Xavier Morin, Auguste Genovesio

**Affiliations:** Scientific Center for Computational Biology, Institut de Biologie de l’Ecole Normale Superieure, CNRS-INSERM-ENS, PSL Research University, 46, rue d’Ulm, Paris, 75005 France; Division cellulaire et neurogenèse, Institut de Biologie de l’Ecole Normale Superieure, PSL Research University, 46, rue d’Ulm, Paris, 75005 France

**Keywords:** Image analysis, Gaussian mixture, High throughput, Mitosis, Time-lapse microscopy, Cell detection

## Abstract

**Background:**

Cell culture on printed micropatterns slides combined with automated fluorescent microscopy allows for extraction of tens of thousands of videos of small isolated growing cell clusters. The analysis of such large dataset in space and time is of great interest to the community in order to identify factors involved in cell growth, cell division or tissue formation by testing multiples conditions. However, cells growing on a micropattern tend to be tightly packed and to overlap with each other. Consequently, image analysis of those large dynamic datasets with no possible human intervention has proven impossible using state of the art automated cell detection methods.

**Results:**

Here, we propose a fully automated image analysis approach to estimate the number, the location and the shape of each cell nucleus, in clusters at high throughput. The method is based on a robust fit of Gaussian mixture models with two and three components on each frame followed by an analysis over time of the fitting residual and two other relevant features. We use it to identify with high precision the very first frame containing three cells. This allows in our case to measure a cell division angle on each video and to construct division angle distributions for each tested condition. We demonstrate the accuracy of our method by validating it against manual annotation on about 4000 videos of cell clusters.

**Conclusions:**

The proposed approach enables the high throughput analysis of video sequences of isolated cell clusters obtained using micropatterns. It relies only on two parameters that can be set robustly as they reduce to the average cell size and intensity.

## Background

Mitosis, the eukaryotes division, is a complex cellular process involving multiple proteins. In multicellular organisms, the precise orientation of cell divisions relative to their environment plays a crucial role in the development, growth, and homeostasis of many tissues [1]. For example, divisions within the plane of epithelial structures contribute to the expansion of the tissue surface and to the maintenance of the epithelial monolayer organization [2], while divisions perpendicular to the epithelial plane contribute to tissue stratification, binary fate decisions and regulation of stem cell pools [3, 4]. Defective control of spindle orientation may be a step in the transformation process leading to cancer [5, 6]. In vertebrate cells, multiple molecular pathways contribute to spindle orientation in response to a variety of stimuli that include intrinsic cell polarity, adhesion to the extracellular matrix, and contacts with their neighbors [1]. Remarkably, these mechanisms are shared by cells grown in a culture dish, and in vitro studies in adherent cells have contributed a lot to our current understanding of spindle orientation.

The aim of the biological study, for which the following development was set, is to identify new regulators involved in the orientation of cell division through a mid-throughput RNAi screen in vitro. To this end, we have developed a specific model of oriented cell division between pairs of cells grown on adhesive micropatterned disks. The precise molecular design of this spindle orientation assay is beyond the scope of the current study and will be described elsewhere, in combination with the results of the RNAi screen (di Pietro et al. in preparation). Here, we present the image analysis approach that we designed with the aim to automatically 1) identify events of cell divisions and 2) measure their orientation relative to their neighbors. Cell culture on micro-patterned surfaces is increasingly used in cell and developmental biology studies using single [7–9], pairs [10], or larger groups of cells [11, 12], owing to the possibility that micropatterning offers to control numerous parameters of the cells environment and therefore reduce intercellular variability. Hence the proposed method for the first step can be generally useful to the parallel study of any event of interest arising in a growing cluster of cells.

**Fig. 1:**
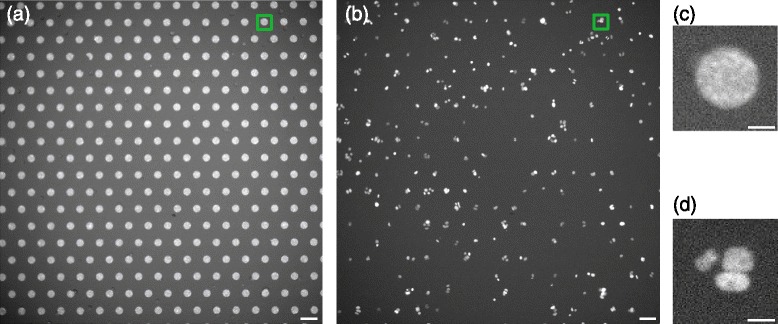
Large series of cell cluster acquisitions using Fibronectin micro-patterns. **a** shows an image displaying all micro pattern positions of a given field of view. This image is captured once at the beginning of the sequence to locate cell patterns. **b** shows an acquisition of one time frame of the H2B-mCherry signal for the same field of view. This image contains the cell clusters. **c** shows one of the pattern position (corresponding to the *green* square on the top right of the field of view in image (**a**) and (**d**) shows the corresponding cell cluster located on it. A movie is automatically extracted from each pattern positions containing cells. The thousands of movies extracted this way from multiple fields of view are then analyzed using the proposed method. Scalebars are 80 *μ* *m* for (**a**) and (**b**) and 20 *μ* *m* for (**c**) and (**d**)

Human cells (HeLa cells) genetically modified to express the H2B-mCherry chromosomal fluorescent reporter were seeded onto thousands of 30 *μm* diameter micropatterned disks coated with fibronectin [13] and imaged over 60 h every 7 min using fluorescence time-lapse microscopy. The honeycomb regular spacing of the adhesive fibronectin patterns, microprinted on a cytorepellent surface, enabled to obtain hundreds of isolated growing clusters of cells per condition (see Fig. 1).

**Fig. 2:**
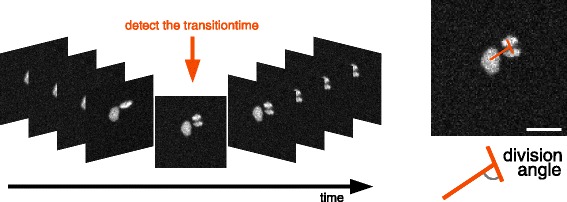
Goal. Automated identification of the first frame containing three cells in the video and computation of the division angle on this frame. Scalebar is 20 *μ* *m*

**Fig. 3:**
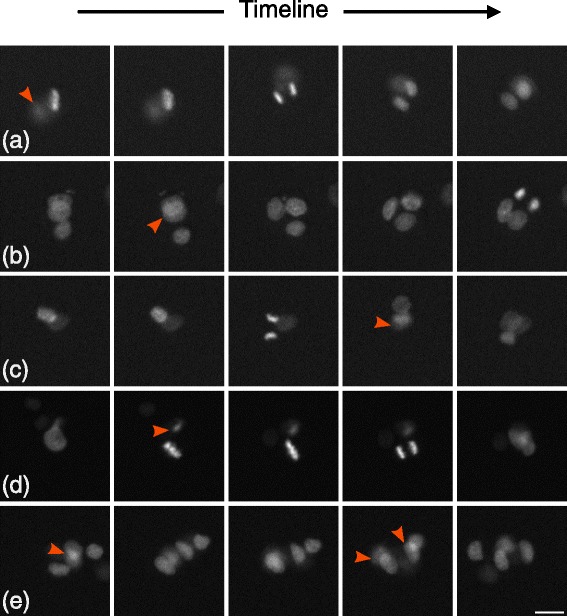
Difficulties. Cell number and location in a packed cluster cannot be robustly assessed with known methods and even sometimes by human vision. Each row shows 5 consecutive frames of a video example that illustrates the variety of difficulties this assay presented. **a** a frequent case where one of the cell is out of focus, (**b**) another frequent case where cells are overlapping, (**c**) a case showing both overlapping and out of focus cells, (**d**) a case where a cell enters the field of view just before mitosis, (**e**) another case showing overlapping cells. Scalebar is 20 *μ* *m*

The development of scripts to detect all pattern positions and extract all single cluster video sequences is fairly straightforward. The purpose of this paper is not to describe this process but rather how we resolved unexpected difficulties inherent to the large variety of cell cluster sequences we had to deal with in the next step of the process. We seek to detect, for each of those sequences, the precise time point when a cluster switches from two to three cells in order to measure the division angle of the occurring division versus the axis formed by the previously existing two cells (see Fig. 2). Hence, only patterns with one cell or two cells at the beginning of the experiment are of interest; however the cell seeding process results in patterns without any cell (which can easily be discarded from the analysis), and patterns with more (3 or more) cells than required, which are therefore densely packed on the pattern. Despite the fact that this description sounds rather simple, in practice, we faced a variety of challenges (see Fig. 3) that made this operation intractable with the most advanced and popular cell detection methods currently available.

For low throughput microscopy image analysis, a variety of semi-automated methods were proposed and are currently largely used to detect cells [14]. By semi-automated we mean an imaging throughput that is low enough (a few images or videos) for manual intervention to help or correct the detection. An exhaustive description of those available semi-automatic methods is out of the scope of this paper. However, as soon as full automation is required because of the throughput, the number of concretely working methods shrink to a few and require the data to meet with some strong hypotheses. One of those hypotheses is that cells must contain a single nucleus [15]. Another important hypothesis that is often made is that nuclei can touch each other but should not overlap [16]. Eventually, the accurate monitoring of topological changes, that is tracking splitting objects over time, highly relies on the accuracy of the cell identification process at each time frame.

Despite fruitful debates about the capabilities of levelset methods to uncover the topological changes in a group of objects to detect at low throughput [17], methods currently used at high throughput for cells detection are rarely based on those approaches because of their lack of robustness in a fully automated process. Instead, the cell detection relies most often on two steps: seed identification followed by segmentation [18–20]. The identification step consists in defining a seed for each object and the segmentation step consists in applying a region growing algorithm initialized by those seeds to uncover objects boundaries. An example of naive approach to seed detection is the local maxima detection after smoothing, which is heavily used at high throughput because of its simplicity, its speed and its robustness for many cell based applications. Regarding the detection step, seeded watershed and coupled explicit or implicit active contours can be used [21–24]. The former methods are currently common practice and proved to be very efficient in detecting millions of regular cells in monolayer where nuclei do not overlap [25] while the later are more rarely seen in practice because of their inherent instability. However the whole process depends primarily on the identification step. That is, the results tend to significantly degrade when nuclei overlap with one another and that seed cannot be correctly identified (see Fig. 3). This is precisely the problem we ran into while using micropatterns.

In the literature, those small fibronectin patterns have mostly been used for experiments with a single cell per pattern (a few exceptions with two cells or more do exist but the pattern makes the position of cells obvious and non overlapping [7, 10]). Moreover, most of the studies were not dynamic and focused on getting reproducible cell shape in order to quantify cytoskeleton organization [26]. Therefore, with a few exceptions, tracking cells on single micropatterns has not yet been an issue using this technology.

In our experiment, the chosen pattern is a disk and the number of cells growing onto them is variable and unknown. Furthermore, the pattern introduces physical constraints that tend to pack cells together as they are dividing, making their individual detection or even a simple counting often difficult (see Fig. 3). Indeed when more than two cells are present on a pattern, their shape differ from cells duplicating freely on an unbounded fibronectin slide. Consequently, nuclei shape and distances between nuclei are impacted. Furthermore, when clusters contain three or more cells, they often overlap with each other, making the detection intractable with previously cited methods. We therefore had to propose a new way to extract information from those packed clusters of cells.

In order to detect in each sequence the first frame showing three cells, our approach consisted in modeling the cell cluster by Gaussian Mixture Models. Hence, a selection process based on the sequence would allow us to determine the number of cells and their positions at each frame. Since the event we were looking for in our study was the second mitosis (that is when one of the two cells divides in a cluster of two cells only), we proposed to fit two hypothesis models to the cell cluster at each time frame: a 2-component and a 3-component 2D Gaussian mixture models (GMM). Fitting a GMM to count and detect biological objects in microscopy images was proposed in the past mostly to model small fluorescent spots or on static images. Thomann et al. [27] used a 3D Gaussian model to approach the point spread function and detect the number of spots reaching super-resolution. A *χ*^2^ test was then used to choose the right number of Gaussians in the Gaussian mixture. However, the number of degrees of freedom of the *χ*^2^ test was defined as the number of pixels lying on the object (a few in the case of spots) which would be unrealistic in our case. Other methods are based on mutual information [28] or are dedicated to mitosis detection in histopathology images [29] but they gave poor results on our data because the cells are more densely packed on micropatterns. However, a close approach was proposed in [30] where numerous cells are tracked in 3D using GMM. The difference with our approach lies in the fact that because the throughput is much higher in our case, images could not be acquired in 3D. Therefore, unlike in 3D imaging, the view is incomplete and cells can overlap with each other and appear out of focus which are the major issues we had to deal with (see Fig. 3).

## Method

**Fig. 4:**
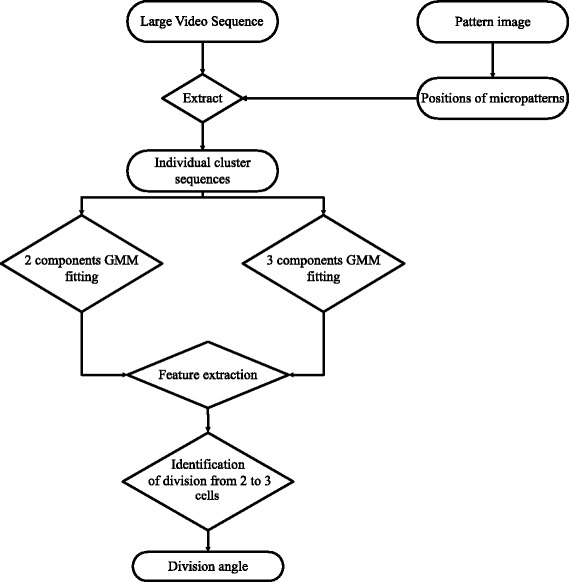
Flowchart of the proposed approach

The proposed approach is composed of four steps described in this section. The first step consists in localizing the fibronectin patterns and cropping the whole video at those locations to obtain individual cluster sequences, the second step consists in fitting 2- and 3-components Gaussian Mixture Model (GMM) onto each frame of each video sequence and the third step consists in the identification of the first frame containing three cells (the transition from 2 cells to 3 cells) using the fitting error difference and other features computed from the GMM parameters. The final step consists in the computation of the angle of division in the identified frame. The whole proposed approach is illustrated in Fig. 4 (and the code is freely available at https://github.com/biocompibens/livespin).

### Extraction of individual sequences from a video

Figure 1 shows the pattern image obtained at the beginning of the sequence acquisition. Each bright area in the pattern image is a micropattern possibly containing an individual and isolated cell cluster. We name a cell cluster a set of cells close to each other that mostly originate from a single cell. Figure 1 also shows a random frame of the video sequence of the H2B-mCherry signal corresponding to the same field of view. Each condition of a screen will be made of two such acquisitions. Since each cell cluster is independent from the other, we set up a system to automatically crop a window around each micropattern over time, thus producing one video sequence per micropattern with a possible cluster on it (see Fig. 1).

In order to take into account the illumination bias (on Fig. 1, intensity at the center of the image is brighter than around the borders) we applied an adaptive equalization of the histogram [31]. Once corrected, the pattern image is fairly easy to segment and a smoothing followed by a cropping around local maxima was sufficient to obtain hundreds of cropped movies, each containing one micropattern location as shown by Fig. 1. From this point, those movies could be analysed independently with the following proposed method.

### Characterization of cell nuclei by Gaussian mixture model

#### GMM as a cell cluster model

**Fig. 5:**
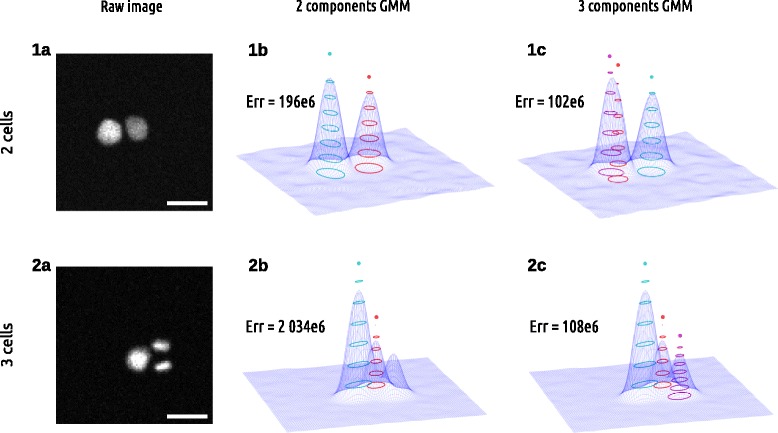
Gaussian Mixture Model fit on cell images. Each row shows an image of cells and the corresponding GMM fit with 2 and 3 component. The first row shows an image with 2 cells while the second row shows an image with 3 cells. The 3-component model (**1c** and **2c**) is always more accurate on any given image than the 2 component model (**1b** and **2b**) but the fitting error difference between the two models can vary significantly. See fitting error *Err* on three cells of the 2-component model (**2b**). We take advantage of this variation over time to detect the transition between 2 cells and 3 cells whether they appear distinct as on this example or they overlap. Scalebar is 20 *μ* *m*

Nuclei of cells expressing H2B-mCherry and imaged via fluorescence microscope exhibit an ovoid structure which can be approximated by a 2D Gaussian distribution of grey level intensity around its center, as shown in Fig. 5. Therefore, an image containing *N* cells could in principle be modelled reasonably well by a Gaussian mixture model (GMM) with at least *N* components. The final goal of the study is to measure the variation of the orientation of the cell division when a cluster goes from two to three cells. Thus our approach consists in comparing the relative quality of reconstruction of the observed cluster by two GMM models with two and three components. This would allow for resolution of both the number of cells and also their positions provided by the model.

In theory, whatever the signal, more components in a GMM leads to a better reconstruction. It is therefore not possible to directly compare the fitting residuals obtained by the two models as the 3-component model would always show a lower error. This model selection issue was discussed in general in the litterature and universal criteria for model selection were proposed in the past as the Akaike Information Criterium (AIC) [32] or the Bayesian Information Criterium (BIC) [33]. Our experience using those criteria independently at each time frame of the sequence led to a totally erroneous identification of the correct cell division frame. We therefore took a different approach as we describe further. However, prior to discussion on model selection, we describe how an accurate fit of the two GMM with two and three components could be achieved at high throughput: that is, on each of the 400 frames of each of the thousand individual movies of cluster we extracted.

#### Fitting the model to the data

The formulation of a 2D Gaussian mixture we used for fitting is the following:

(1) $$ f(\boldsymbol{x}, \boldsymbol{\Theta}_{K})= \sum_{k=1}^{K} w_{k} e^{-\frac{1}{2}(\boldsymbol{x}-\boldsymbol{\mu}_{k})'\boldsymbol{S}_{k}^{-1}(\boldsymbol{x}-\boldsymbol{\mu}_{k})}  $$

where *K* is the number of components of the mixture, *w*_*k*_ is a scalar value indicating the weight (or the intensity at the peak) of the component *k*, ***μ***_*k*_=(*μ*(*x*),*μ*(*y*))^′^ is the 2D location of the component *k* in the plane and ***S***_*k*_ is its covariance matrix that reads:

(2) $$ \boldsymbol{S_{k}}=\left[ \begin{array}{ll} {\sigma_{1}^{2}} & \sigma_{12} \\ \sigma_{12} & {\sigma_{2}^{2}} \end{array}\right]  $$

So each component is fully characterized by a set of 6 parameters *P*_*k*_={*w*,*μ*(*x*),*μ*(*y*),*σ*_1_,*σ*_2_,*σ*_12_} and the concatenated set of parameters ***Θ***_*K*_={*P*_1_,…,*P*_*K*_} fully characterizes a *K* components mixture. Following the two hypotheses model with two and three components we are interested in testing, we build GMMs with 12 or 18 parameters respectively. We use the Powell algorithm [34] to minimize the least-square residual between a frame image *I* and the *K* component GMM image model *M*_*K*_ that reads: $f_{\text {err}} = \sum _{x, y} \left [I(x,y) - M_{K}(x,y)\right ]^{2}$.

#### Parameters initialization

One of the main difficulties in minimizing such a residual is that given the large number of parameters (12 or 18), the convergence toward the global minimum is not systematic. In order to ease this convergence, it is therefore crucial to set the initial parameters with values close to the optimal solution.

For the first image of the sequence, we take advantage of the fact that an average nucleus diameter $\bar {d}_{\text {nuc}}$ and intensity $\bar {w}_{\text {nuc}}$ can be easily estimated from the data. As $\bar {d}_{\text {nuc}}$ can be modeled as the Full Width at Half Maximum [35], we first define a 2D Gaussian kernel with $\bar {\sigma }_{\text {nuc}}=\bar {d}_{\text {nuc}}/(2\sqrt {2ln(2)})$. Local maximas are then detected on an image smoothed by this kernel and limited by a foreground defined with the Otsu method [36]. Those local maximas are then used as initial values for Gaussian component locations. If the number of detected maxima is lower than the number of components of the model (e.g. when cells overlap), then additional random locations on the foreground are added. The intensity $\bar {w}_{\text {nuc}}$ is directly used to initialize *w*. The remaining parameters *σ*_1_, *σ*_2_ and *σ*_12_ are initialized with median values of a set of previously fitted GMM components with random initialization.

For the rest of the frames in the sequence, parameters are initialized with values obtained from the fitting at previous frames and from observations obtained from the current frame. In two consecutive frames with no mitotic event (that is in the large majority of the cases), the position, the intensity and the shape of the cells are not supposed to change much given the time interval between video frames (in video duration of 7 min). Therefore, the parameters *μ* and *w* could be initialized on the next frame by the values obtained for the same parameter at the last frame. This would read $\hat {\boldsymbol {\mu }}_{t}=\boldsymbol {\mu }_{t-1}$ and $\hat {w}_{t}=w_{t-1}$. However, in the case where mitosis happens, the location and the intensity of some of the cells suddenly change. To take into account this event, local maxima of the image are also precomputed on each image and the locations (resp. the intensity) of each component are initialized by a value half way between the location (resp. the intensity) obtained at the previous frame and the location (resp. the intensity) of the closest local maxima possibly detected on the current frame. This reads $\hat {\mu }_{t}=(\mu _{t-1} + {\mu ^{D}_{t}})/2$ and $\hat {w}_{t}=(w_{t-1} + I({\mu ^{D}_{t}}))/2$ where ${\mu ^{D}_{t}}$ is the location of the closest detected maxima on frame *t*. This simple method ensures that the fitting process will be initialized a priori as close as possible from the optimal solution while it is not known if a mitotic event occurs or not.

Furthermore, we observed that while the shape of a nucleus is not changing much between two consecutive frames (except at a mitotic event time), on the contrary its orientation is quite dynamic (cells are often rotating). Therefore, we decided to uncouple the shape and the orientation of each component at each time frame in order to properly initialize the fitting process on the next frame for each of those parameters. In the formulation we use, shape and rotation are mixed into the covariance matrix. By diagonalizing the covariance matrix ***S***_*k*,*t*−1_ of each component *k* resulting from the previous frame we obtain *λ*_1_ and *λ*_2_ the eigenvalues corresponding respectively to the length of the major and the minor axes of the ellipse and the corresponding eigenvectors ***v***_***1***_ and ***v***_***2***_ from which the angle of the ellipse’s major axis can be computed: $\theta _{k,t-1}=\arctan (v_{1}(y)/v_{1}(x))$.

When the nucleus rotates, solely the angle *θ* varies, not the shape represented by *λ*_1_ and *λ*_2_. Therefore, we proposed an initialization of the angle to be a linear extrapolation of the two previous frames (constant speed rotation) with *δ*_*k*,*t*−1_=*θ*_*k*,*t*−1_−*θ*_*k*,*t*−2_ leading to the following rotation matrix:

(3) $$ \boldsymbol{\hat{R}_{k,t}}=\begin{bmatrix}cos(\delta_{k,t-1})& -sin(\delta_{k,t-1})\\ sin(\delta_{k,t-1}) & cos(\delta_{k,t-1})\end{bmatrix}  $$

Eventually, the covariance matrix containing the parameters $\hat {\sigma }_{1}, \hat {\sigma }_{2}$ and $\hat {\sigma }_{12}$ is initialized by rotating the covariance matrix obtained at previous frame the following way:

(4) $$ \boldsymbol{\hat{S}_{k,t}}=\boldsymbol{\hat{R}}_{k,t}\boldsymbol{S}_{k, t-1}\boldsymbol{\hat{R}}_{k,t}^{-1}  $$

#### Constraints to ensure convergence

As our model includes 12 parameters in the case of 2 components and 18 parameters in the case of 3 components, even with a precise initialization the fitting process may diverge (e.g. one component may easily collapse or move outside the frame). We enforced the convergence by adding penalty terms to our error function.

The first penalty term concerns the locations ***μ***_*k*_ of the Gaussian components. A reasonable hypothesis made on those locations is that they should lie onto the intensity foreground. Therefore, we computed a distance matrix *D* which is the size of the image. Each position of *D* maps to 0 inside the foreground and to the distance to the closest foreground pixel outside the foreground. In order to prevent the Gaussian components to move away from the foreground we use this matrix in the following penalty term that rapidly increases the error when a component location moves away from the foreground:

(5) $$ f_{\text{loc}} = \sum_{k=1}^{K} D(\boldsymbol{\mu}_{k})^{2}  $$

The second penalty term concerns the area of the nuclei that we know is about a given value $\bar {A}_{\text {nuc}}=\pi \bar {d}_{\text {nuc}}^{2}/4$ entirely defined by our prior estimation of $\bar {d}_{\text {nuc}}$. It ensures that the final area of the component represented by the determinant of the covariance matrix is not exaggeratedly different from this given area and it reads:

(6) $$ f_{\text{vol}}=\sum_{k=1}^{K} (|\boldsymbol{S}_{k}|-\bar{A}_{\text{nuc}})^{2}  $$

The last penalty term concerns the intensity of the nucleus that should not collapse and that we know is about a previously defined $\bar {w}_{\text {nuc}}$. Indeed, we observe that without this term, one of the components could easily end up modeling the background. It reads:

(7) $$ f_{\text{int}}=\sum_{k=1}^{K} (w_{k}-\bar{w}_{\text{nuc}})^{2}  $$

The global error, now penalized by those terms, reads:

(8) $$ f_{\text{global}} = f_{\text{err}} \cdot \big(1 + f_{\text{loc}} + f_{\text{vol}} + f_{\text{int}} \big)  $$

Note that each of those additional constraints prevents the optimization process to move toward absurd values by artificially increasing the total error outside an acceptable range. Therefore, they drastically modify the objective function outside an acceptable range of parameter values while they preserve the function within this range. The consequence is that they do not modify significantly the minimum of the function.

### Time features computed from the GMMs

At this stage, large sets of data can be fully automatically processed by extracting all single pattern videos and automatically fitting a 2-component GMM and a 3-component GMM on each of their time frames. Two parameters only need to be set: the approximated nuclear diameter $\bar {d}_{\text {nuc}}$ and intensity $\bar {w}_{\text {nuc}}$. Those values can be easily recovered.

In order to identify the first frame onto which three cells can be observed (that is right at the second division) on each of those videos, we propose to compute the derivative over time of three features. Those features are the fitting error ratio between both models, the minimum distance between the three component centers and the variance of intensity between the closest component centers. None of those require any parameter and they are described below.

#### *F*_1_: fitting error ratio

**Fig. 6:**
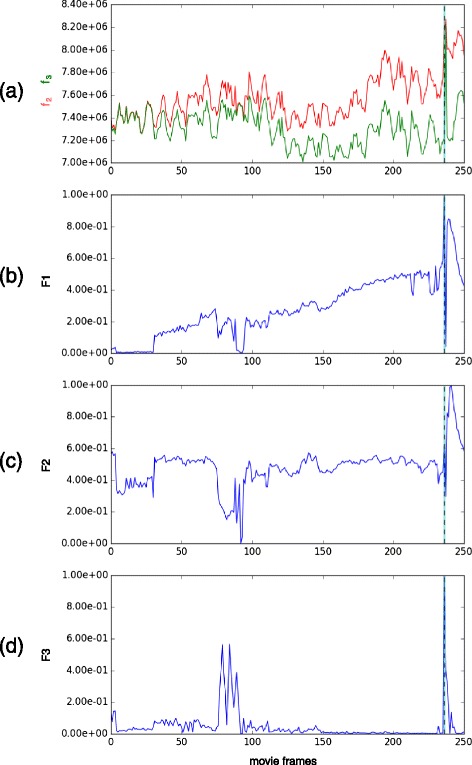
Time features *F*1, *F*2 and *F*3 on an example video. The *dashed vertical line* indicates the event of interest we are seeking to identify when a third cell appears. **a** residual *f*2 of the 2-component model in *red*, *f*3 of the 3-component model in *green* and (**b**) *F*_1_, their ratio. **c** *F*_2_, the distance between the two closest centers of the 3-components model. **d** *F*_3_, the variance of the intensity values between the two closest centers of the 3-component model

**Fig. 7:**
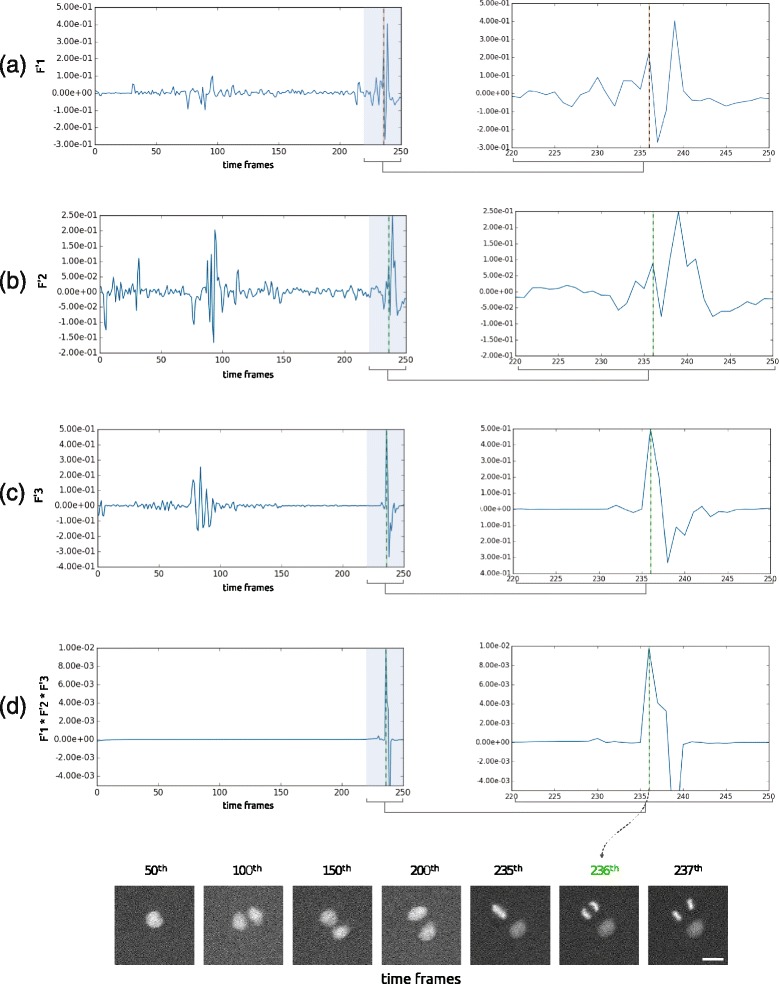
Derivatives of the time features (corresponding to the example given in Fig. 6) (**a**) *F*1′, (**b**) *F*2′, (**c**) *F*3′ and (**d**) their product over time. The right panels are zooms in the peak region. A first clear peak of the feature derivatives product can be observed at the frame of interest. Scalebar is 20 *μ* *m*

We are interested in finding a specific anaphase event: the first frame onto which three objects can clearly be identified (see Fig. 2). In theory, a GMM with three components (residual *f*_3_) should always fit better to the signal than a GMM with two components (residual *f*_2_). This is illustrated on a single image by Fig. 5 and on a whole sequence by Fig. 6a where *f*_3_ is constantly lower than *f*_2_. However, our rationale is that the transition time from two to three nuclei will be the moment when the residual ratio between both GMM fitting suddenly becomes significantly higher. Such a pattern can be observed from the derivative over time of the residual ratio *F*_1_(*t*)=*f*_3_(*t*)/*f*_2_(*t*) across the entire sequence right when this event is happening (see Fig. 7a and b).

#### *F*_2_: distance between the closest components

As shown in Fig. 6c, the distance between the two closest centers in the 3-component model $F_{2}(t)=\min \{\|\mu _{i}(t)-\mu _{j}(t)\|_{2}, \forall (i,j) \in \{1,2,3\}^{2}, i\neq j\}$ becomes much larger when the mitotic event of interest happens. This is because when a 3-component GMM is used to model two cells, one of the cells ends up being modeled by two components and therefore shows two very close centers. However, when one of the cells splits into two, the 3-component GMM correctly models the cluster, and each component matches a single cell. Consequently, the minimum distance between any two centers suddenly increases.

#### *F*_3_: variance of intensity between the closest components

Along with the distance between the closest centers, the intensity variation of the pixels between those two closest centers also provides information. Indeed, if the variance is high, it denotes that both foreground and background pixels were considered in the calculation, while if the variance is low, it means that only foreground pixels were used. Therefore, this feature tends to measure whether or not the two closest components of a 3-component model are separated by some background or not and therefore if they model or not the same cell. The feature *F*_3_ over time for an example cluster can be seen in Fig. 6d.

### Identification of the division time of interest

In order to detect sudden changes over time using the features described above, we compute their derivatives. Hence we search for a sudden peak in those features’ derivatives (see Fig. 7). In practice, there is a large variability of events we have to deal with when processing hundreds of videos of that kind. Using those three features simultaneously increases the ability of the approach to detect the division time of interest. We show on an example (see Fig. 7) and on a larger study (data not shown) that using the product of those features’ derivatives over time allows to extract this event with a better accuracy than using only one or two of them.

### Computation of the division angle

The method described above enables detection of the time of the first anaphase image on a movie with two cells. Detecting the right time is essential in order to measure the correct angle, because cells move and rotate from one time point to the next, especially when there are more than two cells on a pattern. Moreover cells can die or image acquisition can have started when three or more cells were already on the pattern. In those last cases, the error model would not fit. This allows us to exclude sequences where a division angle cannot be measured.

Once the right image is selected, the parameters of the fitting give the positions *μ*_*k*_ and sizes |***S***_*k*_| of the corresponding underlying nuclei (see Fig. 5). From those measures, nuclei issued from the last division are chosen to be the two smallest Gaussian objects. Using those, the extraction of the angle described by the Fig. 2 is straightforward.

## Results

To our knowledge, no available software could provide a full solution dedicated to the type of assay we propose (i.e. an automated tracking of overlapping cells on thousands of individual movies). Therefore, it was not possible to strictly compare our approach to another possibly existing method. However, a freely available software program that could have matched our need was Cellprofiler [25] because in principle, it enables the tracking of cells over time in a large set of image sequences, using the Hungarian algorithm. However, cell detection in Cellprofiler is based on a maxima detection followed by a seeded Watershed segmentation so we expected it not to perform well in detecting overlapping and dividing cells. In accordance, the results we obtained were dramatically poor. A quantitative comparison here would be meaningless, as almost no mitotic event could be identified this way. However, it was possible to compare our automated approach to a large set of data (4000 sequences) that has been exhaustively analyzed by a human tester, and considered thereafter as the “ground truth” for our method.

### Experimental data

The dataset we created to validate the method is made of several videos of hundreds of cell divisions under three biological conditions. Those conditions are as follows: as a negative control, we used an siRNA targeting Cyclophilin, which is proposed as one of several standard negative controls by GE-Dharmacon in their ON-target+ human siRNA libraries. LGN (Leucine-Glycine-Asparagine repeat protein) was used as a positive control: LGN is an adaptor molecule involved in the localized recruitment of dynein motor complexes at the cell membrane, which direct forces exerted on astral microtubules. LGN is a central regulator of spindle orientation in many animal cell types (reviewed in [1]). Our paired-cell assay (di Pietro et al, in preparation) is designed to specifically depend on the “LGN-complex” molecular cascade. siRNA against LGN therefore significantly alters spindle orientation in this assay. The third siRNA targets p62, which is part of the dynactin molecular complex and as such a candidate for the regulation of dynein activity and spindle orientation. It is therefore expected to differ significantly from the negative control, and to yield results similar (but not necessarily identical) to the positive control. As we aim at using this method on a large set of conditions for which we will have a variation in the number of patterns we will obtain per condition, each condition for our test was respectively made of 4, 5 and 6 videos covering each field of view. Each field of view was made of about 250 frames of size 2048×2048 pixels. Figure 1 shows a frame of such a video captured by a wide field fluorescence microscope and containing about 280 patterns (excluding those touching the borders).

The fitting process is the most time-consuming step of the analysis. It takes 2 seconds for each image on a PC with Intel Core i7-4800MQ 2.7 GHz with 16 GB RAM. As analyzing one sequence requires to test two models on 250 frames, the overall process for one cluster containing cells takes 25 min. However, we used a computing cluster to process hundreds of cell clusters simultaneously.

### Precision of the event detection

**Fig. 8:**
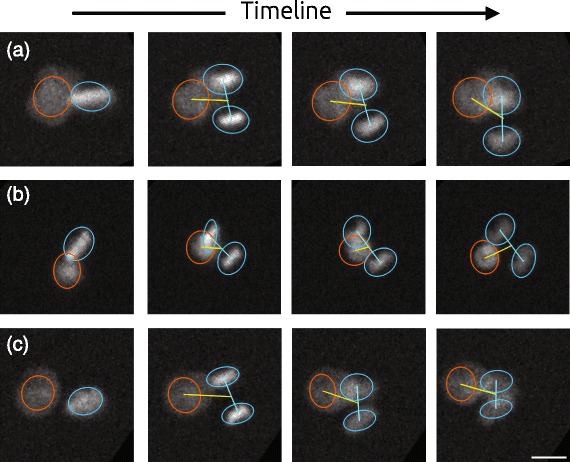
Three examples (**a**, **b** and **c**) of identification of the mitotic events of interest over time. On the three examples, our algorithm succeed to identify the correct number and position of the cells despite frequent overlap and differences in intensities. Scalebar is 20 *μ* *m*

The dataset proposed was subject to a fully manual analysis on one hand and a fully automated analysis on the other hand. In both cases, the goal was to retrieve the sequences containing a transition from two to three cells and the exact time frame of this transition in order to measure the division angle. Figure 8 shows a few examples of those transition events automatically detected. Table 1 describes in detail the pattern and event count along the process. In summary, about 40 % of the pattern contained no cells, 10 % contained obviously too many cells at the beginning of the sequence to be processed further and 50 % were processed further using the proposed analysis to search for a possible transition from two to three cells. Eventually, the manual analysis identified that 15 % of the sequence contained a transition from two to three cells, while the automated analysis only found 10 %. Interestingly, for any condition, at least 80 % of the events found automatically were also part of the event found manually (this could be called the precision as we are confident in our case that our manual analysis is very close to the ground truth). A teddious investigation of the differences between the manual and the automated analysis led to the conclusion that the automated method could sometimes fail in the case where some debris crossed the field of view, in case of dead cells or when two cells divided at the same time to produce four cells. Eventually, the event could also be missed when no clear significant peak arises in the derivative of the feature over time, due to extreme cases of simultaneous out of focus and overlapping.

**Table 1:** Pattern count on raw data (A and B), after a rough preprocessing step (C, D and E) to discard empty patterns or patterns containing obviously more than 2 cells on the first frame of the sequence. Eventually, the number of pattern where a transition from two to three cell was detected automatically (F) or manually (G). While a lower number of event is selected automatically, more than 80 % of the events selected automatically were also part of the manual selection whatever the experimental condition

	Cyclophilin	LGN	p62
	siRNA	siRNA	siRNA
A) Total number of fields of view (=large videos)	4	5	6
B) Total number of micropatterns (=single cluster videos)	1116	1393	1668
C) Micropatterns with no cells (excluded)	400	607	719
D) Micropatterns with too many cells at time 0 (excluded)	51	64	120
E) Micropatterns with a low number of cells at time 0	665	722	829
F) Events selected automatically from (E)	122	135	97
G) Events selected manually	184	197	227
H) Proportion of (F) also in (G)	85.9 %	82.5 %	81.8 %

### Accuracy of the angle distributions

Most importantly, whatever the error rate the algorithm or a human could make, we could assess here that both reach the same conclusion regarding the impact of a perturbation at a 10 % significance level. This can be observed on two statistical analyses. On one hand, in Table 2 a Kolmogorov-Smirnov test cannot reject the hypothesis of similarity between the angle distributions obtained manually and automatically for each condition. On the other hand, Table 3 shows that the comparison between any two pairs of conditions reaches also a similar conclusion: the similarity between distributions is systematically rejected. It should also be noted that while the difference between controls (Cyclophilin vs LGN) is still confirmed by the two approches at a 5 % significance level, the automated analysis seems to remain less accurate than the manual one at detecting a more subtle change in the distribution produced by the siRNA against p62.

**Table 2:** The angle samples obtained from a manual selection or an automated analysis are similar: the null hypothesis of a KS test (“both samples come from the same angle distribution”) cannot be rejected at a 10 % significance level

	Manual (M)		Automated (A)		(M) vs (A)
	median	stdev	median	stdev	KS-test *p*-value
Cyclophilin siRNA	23	30	31	31	0.229
LGN siRNA	71	26	72	25	0.620
p62 siRNA	62	28	54	30	0.246

**Table 3:** The angle distributions obtained from a manual selection and an automated analysis reach similar conclusions: the null hypothesis of a KS test (“both samples come from the same angle distribution”) is rejected for any two couple of conditions at a 10 % significance level

KS-test *p*-value	Cyclophilin siRNA	Cyclophilin siRNA	LGN siRNA
	vs	vs	vs
	LGN siRNA	p62 siRNA	p62 siRNA
Manual	3.22e-15	9.47e-11	5.64e-02
Automated	2.09e-07	1.65e-02	2.72e-04

## Discussion

In order to factor out some possible issues that may have occured we performed additional tests.

### Possible bias induced by the statistical test

**Fig. 9:**
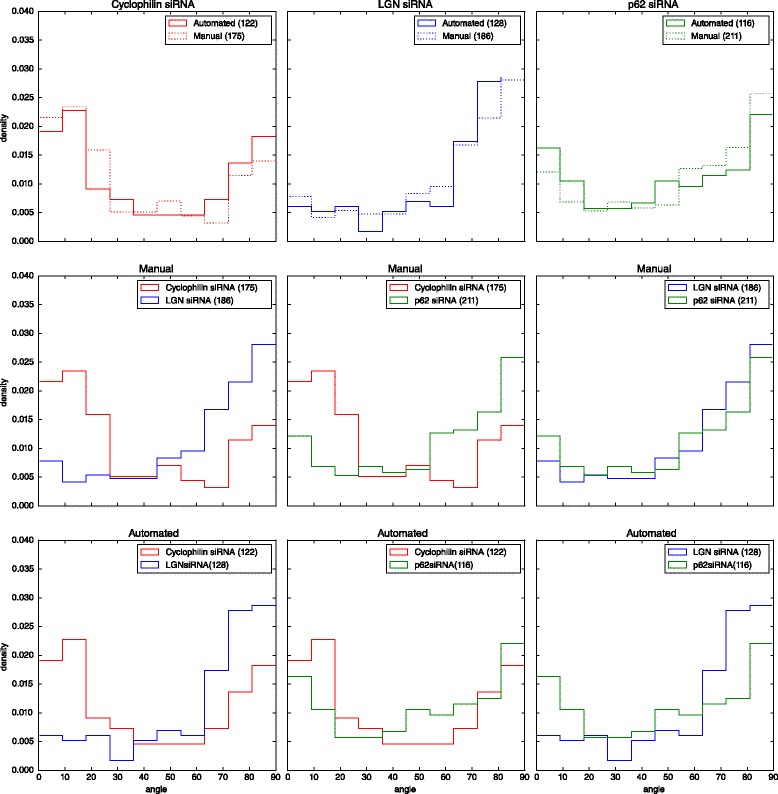
Distributions of angles comparisons. *First row*: manual and automated analyses are plotted against each other for each condition. *Second row*: manual analysis plotted for all couples of conditions. *Third row*: automated analysis plotted for all couples of conditions

**Fig. 10:**
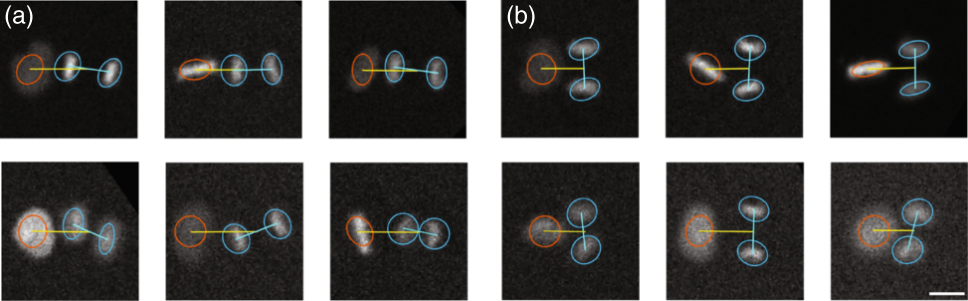
Sorted results. **a** presents 6 events of interest showing cells dividing in alignment with the previous two cells while (**b**) presents 6 other events where the division occurs orthogonally to the previous two cells. Scalebar is 20 *μ* *m*

Interestingly, Fig. 9 shows that the distributions of angles we obtained were not mono-modal or Gaussian-like as we may have expected, but rather bimodal (extreme case examples of those two phenotypes could be retrieved from the automated analysis, see Fig. 10). In order to take into account this, statistical tests known to be more sensitive to the sides of a distribution, such as the Anderson-Darling test, were also tried but they reached very similar conclusions (data not shown).

### Possible bias produced by the pattern

**Fig. 11:**
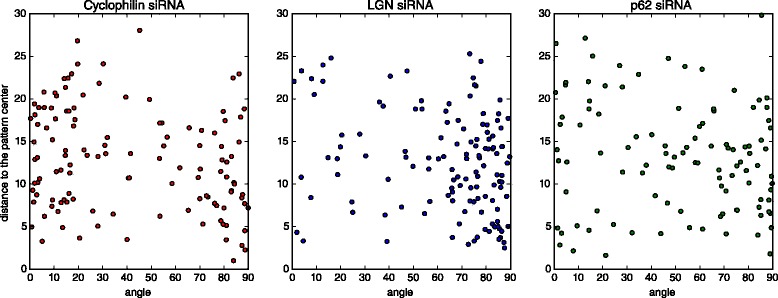
The location and the angle of a mitotic event are not correlated. For each condition we plot the angle found for each sequence versus the distance from the mitotic event to the center of the pattern. Those plot show that there is no correlation between the position where the angle was measured on the pattern and the value of this angle

As the pattern’s edge forms a barrier and the pattern’ size is in the order of the cell size, division is constrained. However, we investigated if there was any relation between the angle and the position of the cells on the pattern (e.g. are cells dividing closer to the edge more likely to divide orthogonally?). The Fig. 11 shows that the position on the pattern has no effect on the angle.

## Conclusion

In this paper we proposed a high throughput method to automatically detect the transition of a cell cluster from two to three cells in thousands of videos. The proposed algorithm performs a robust implicit tracking of cells even when they are packed, overlap or are not clearly distinguishable. The approach is based on a robust fitting of two-dimensional Gaussian mixture models with two and three components on each frame of the video. We showed that the derivatives of the residual ratio between the two models, the distance between the two closest centers and the variation of intensity between them was sufficient to detect the exact time of an event of interest. We showed, using three independent conditions, that the distributions of angles obtained automatically were very similar to those obtained through a very tedious manual annotation that took several days and would be impossible to concretely extend to hundreds of conditions. While the focus of our study was to monitor the division orientation, the same principle can easily be extended to many other questions through the calculation of other features obtained using the proposed approach.

## Availability of data and materials

All code and data necessary to reproduce the results of this paper is freely available on GitHub

- **Project name:** livespin
- **Project home page:**https://github.com/biocompibens/livespin
- **Archived version:**https://github.com/biocompibens/livespin.git
- **Operating system(s):** Platform independent
- **Programming language:** Python
- **Other requirements:** Python 2.7
- **License:** GNU GPL 3.0
